# A new kind of membrane-tethered eukaryotic transcription factor that shares an auto-proteolytic processing mechanism with bacteriophage tail-spike proteins

**DOI:** 10.1242/jcs.133231

**Published:** 2013-11-15

**Authors:** Hiroshi Senoo, Tsuyoshi Araki, Masashi Fukuzawa, Jeffrey G. Williams

**Affiliations:** 1 Department of Biology, Faculty of Agriculture and Life Science, Hirosaki University, Hirosaki, Aomori 036-8561, Japan; 2 College of Life Sciences, Welcome Trust Building, University of Dundee, Dow Street, Dundee DD1 5EH, UK

**Keywords:** *Dictyostelium*, Transcription factor, Membrane tether, Myelin-gene regulatory factor (MRF), Bacteriophage, C-terminal intramolecular chaperone domain (CIMCD), Serine-lysine dyad

## Abstract

MrfA, a transcription factor that regulates *Dictyostelium* prestalk cell differentiation, is an orthologue of the metazoan myelin gene regulatory factor (MRF) proteins. We show that the MRFs contain a predicted transmembrane domain, suggesting that they are synthesised as membrane-tethered proteins that are then proteolytically released. We confirm this for MrfA but report a radically different mode of processing from that of paradigmatic tethered transcriptional regulators, which are cleaved within the transmembrane domain by a dedicated protease. Instead, an auto-proteolytic cleavage mechanism, previously only described for the intramolecular chaperone domains of bacteriophage tail-spike proteins, processes MrfA and, by implication, the metazoan MRF proteins. We also present evidence that the auto-proteolysis of MrfA occurs rapidly and constitutively in the ER and that its specific role in prestalk cell differentiation is conferred by the regulated nuclear translocation of the liberated fragment.

## Introduction

Despite its essential role in vertebrate brain function the process of myelination within the central nervous system (CNS) is poorly understood at the molecular level. Recently, however, a transcription factor has been described that is required for the expression of a large number of mouse CNS myelin genes (Emery et al., 2009). Overexpression of myelin gene regulatory factor (MRF), stimulates transcription of CNS myelin genes and mice lacking MRF in the oligodendrocyte lineage are defective in myelination. *Dictyostelium* MrfA is an orthologue of MRF (Senoo et al., 2012). It was identified as the transcription factor that activates expression of a reporter construct which defines a subtype of stalk cell precursors. These cells, the pstA cells, comprise the anterior half of the prestalk region and can be recognised by their ability to utilise a cap-site proximal region of the *ecmA* promoter (Early et al., 1993). Multimerisation of an essential 39 nt distal subfragment of this region, and insertion next to basal promoter elements, generated a reporter construct that directs pstA-specific gene expression (Senoo et al., 2012). Combinatorial mutations in the three MrfA binding sites within the 39-mer inactivated their *in vitro* binding, but mutation of any single site was sufficient to eliminate reporter expression. When transformed into an *mrfA* null strain, the unmutated multimer construct was also inactive.

MrfA and the metazoan MRFs contain a region of homology to the DNA-binding domain of the yeast Ndt80 protein and an additional region of homology that we termed the MRF domain. We now demonstrate that MrfA and animal MRFs also contain a predicted transmembrane (TM) domain: the defining feature of membrane-tethered transcription factors. The first membrane-tethered transcription factors to be described were two bZIP proteins that are sequestered in the ER and the Golgi: SREBP-1 and SREBP-2 (Brown et al., 2000). When cellular sterol concentrations fall they are activated by the sequential action of two proteases. Both SREBP-1 and SREBP-2 contain two TM domains and one of the two proteases cleaves in the lumenal loop that separates them. Then, the second protease liberates the bZIP domain, by cleavage within the TM domain. The bZIP-domain-containing fragment migrates to the nucleus and activates expression of genes involved in cholesterol uptake and biosynthesis.

Several other transcriptional regulators use a similar activation mechanism, including ATF6 and Notch (Haze et al., 1999; Weinmaster, 2000; Ye et al., 2000). ATF6 uses the same two proteases for processing as the SREBPs whereas Notch uses two different enzymes. Again, however, one of the two Notch proteases catalyses Notch intramembrane proteolysis. This general form of processing is therefore known as RIP (regulated intramembrane proteolysis). There is also another, less intensively investigated, form of regulated processing, known as RUP, where cleavage occurs outside the TM domain and results from ubiquitin-proteasome-dependent processing (Hoppe et al., 2000).

Here, we show that MrfA, and by bioinformatic implication, the animal MRF proteins, are processed by a totally different cleavage mechanism from either RIP or RUP. This autocatalytic mechanism was previously only documented for bacteriophage tail-spike proteins (Schwarzer et al., 2007). These are synthesised with a C-terminal extension, the C-terminal intramolecular chaperone domain (CIMCD). The CIMCD facilitates trimerisation of the protein and is then cleaved off in an auto-proteolytic reaction that uses a catalytic serine-lysine dyad (Schulz et al., 2010).

## Results

### The MRF-like proteins contain a TM domain

**Fig. 1.**
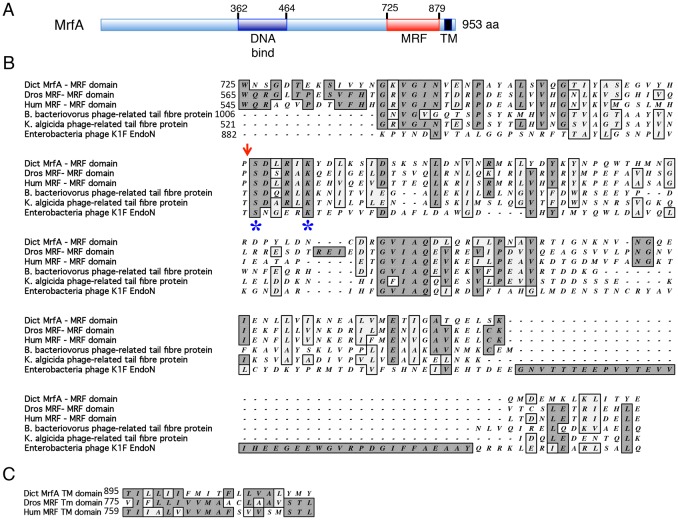
**MrfA and related sequences.** (A) Conserved domains within MrfA. Schematic representation of MrfA showing the positions of the regions conserved with metazoan MRFs. Most of the unassigned sequence is the translation product of AAC repeats (Senoo et al., 2012). (B) Alignment of MRF domains with CIMCDs. The alignment was generated using ClustalW on the MacVector sequence analysis package v9.0 (MacVector). The accession numbers for the sequences aligned with MrfA are: Dros (*D. melanogaster*), MRF NP_611893; Hum (*H. Sapiens*), MRF NP_037411.1; *B. bacteriovorus* phage-related tail fibre protein, NP_969368.1; *K. algicida* phage-related tail fibre protein, EDP95693; Enterobacteria phage K1F EndoN, YP_425027. The red arrow shows the sites of cleavage within EndoNF. The blue asterisks mark the serine and lysine residues that make up the autocatalytic dyad. (C) Alignment of the predicted TM domains of MrfA, *Drosophila* and human MRFs. This alignment was generated as in B.

**Fig. 2.**
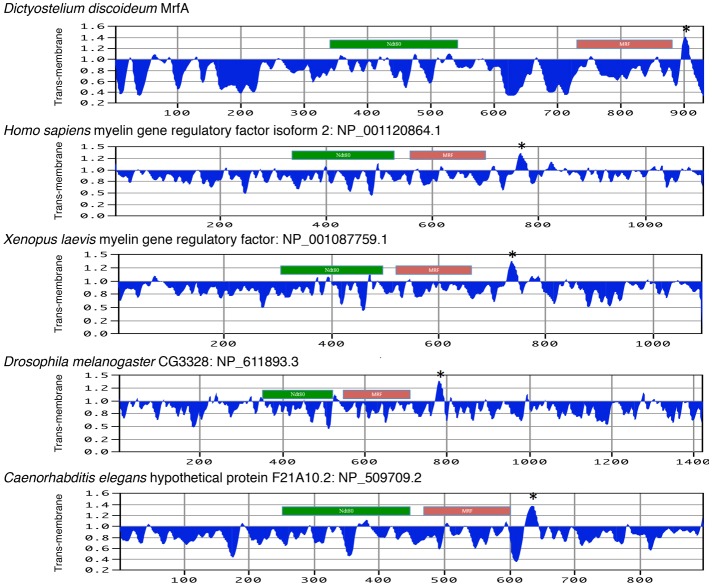
**TM domain predictions for MRF proteins.** TM domain prediction was performed for the indicated proteins using the the ‘Argos’ prediction method on the MacVector sequence analysis package. The asterisked peaks are strongly predicted to be TM domains. Also indicated are the approximate positions of the two regions of primary sequence similarity: the Ndt80 DBD (green) and the MRF domain (red). Results were derived from an NCBI BLAST search performed at default settings using MrfA as the query sequence.

Murine MRF is the founder member of a protein family that was found to be represented in vertebrate and invertebrate animals, but not in fungi (Emery et al., 2009) (Fig. 1A,B). All contain a region with sequence similarity to the DNA-binding domain of yeast Ndt80 and key residues essential for DNA binding of Ndt80 are necessary for MrfA function (Senoo et al., 2012). The defining feature of the family is the presence, proximal to the C terminus, of an ∼150 amino acid region that we designated the MRF domain (Senoo et al., 2012) (Fig. 1A,B). The MRF domain corresponds to the combined form of two more recently assigned ‘pfams’: pfam13384, ‘chaperone of endosialidase’, and close downstream, pfam13887, ‘myelin gene regulatory factor C-terminal domain 1’. We now report that MrfA and animal MRF proteins, from a variety of phyla, also contain a predicted TM domain, situated in a similar downstream position relative to the MRF domain (Fig. 1A,C and Fig. 2).

### Analysis of a doubly tagged form of MrfA reveals a site of proteolyic processing

**Fig. 3.**
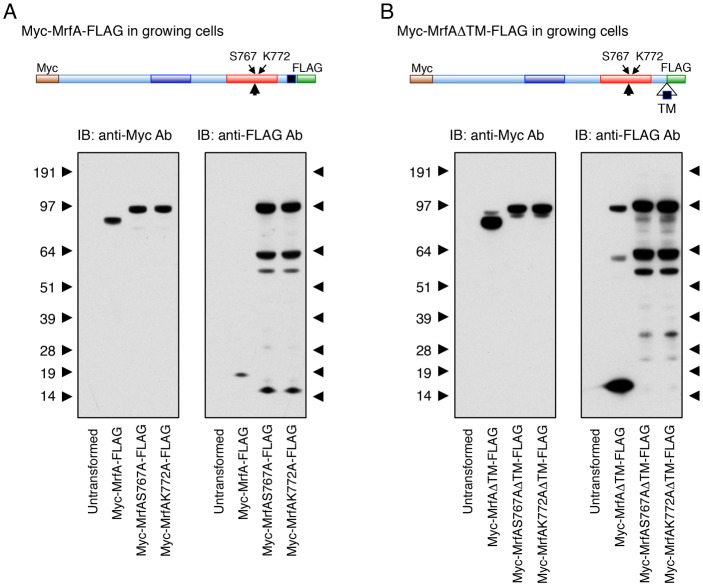
**MrfA processing revealed by western blot analysis.** (A) MrfA and (B) MrfAΔTM with a Myc tag at their N-terminus and a FLAG tag at their C-terminus, were point mutated to alanine at S767 or K772. The resultant constructs were expressed under the semi-constitutive actin-15 promoter in *mrfA* null (*mrfA*^–^) cells. Growing cells were lysed and subjected to western blot analysis using the 9E10 Myc monoclonal antibody or the 1E6 FLAG antibody.

The fact that MrfA contains a predicted TM domain suggests that it is synthesised as a membrane-tethered protein that is released by proteolytic processing. To analyse MrfA processing, a Myc tag was added to its N-terminus and a FLAG tag was added to its C terminus; this generated Myc-MrfA-FLAG. A C-terminal deletion mutant, lacking 38 amino acids that encompass the predicted TM domain, was similarly tagged to generate Myc-MrfAΔTM-FLAG. Both constructs were expressed under control of the semi-constitutive actin-15 promoter and analysed in growing *mrfA* null cells. Western blot analysis of Myc-MrfA-FLAG using a Myc antibody detected a single band of ∼85 kDa (Fig. 3A). This was 20 kDa less than the predicted molecular mass of the full-length protein, and suggests a proteolytic processing event that leaves the N-terminus intact. Analysis of the same samples using a FLAG antibody revealed a band of 19 kDa (Fig. 3A). In combination, the Myc and the FLAG tag results suggest a single cleavage of the 104 kDa precursor within the MRF domain.

Analysing the TM deletion mutant, Myc-MrfAΔTM-FLAG, using the Myc antibody again revealed a predominant band of 85 kDa but now there was a fainter band of higher apparent molecular mass that could be unprocessed, full-length precursor (Fig. 3B). When the same construct was analysed using the FLAG antibody there was an abundant species of about 15 kDa, very close to the expected size for the processed C-terminal fragment (because the TM deletion removes 4.6 kDa from the C terminus of the estimated 19 kDa cleavage fragment). The marked disparity in the amount of FLAG tagged C-terminal cleavage product, between parental and TM deletion forms, suggests that deletion of the TM domain, and presumable exclusion from a membrane compartment, stabilises the peptide in some way. There was also, again, a less-abundant species that migrated at an approximate molecular mass of 100 kDa; the size expected for the full-length Myc-MrfAΔTM-FLAG molecule (the other minor band of ∼60 kDa is presumably a natural or artefactual minor cleavage product). The implication is again that the TM domain is not essential for processing, but its deletion reduces the rate of processing slightly, resulting in low-level accumulation of full-length precursor.

### The MRF domain has sequence similarity to bacteriophage autocatalytic cleavage domains

The cleavage position for MrfA lies in its MRF domain and, within this domain, there is extensive sequence similarity to the CIMCDs of bacteriophage tail and spike fibres (Fig. 1B). These are autoproteolytic cleavage domains (Schwarzer et al., 2007). The crystal structure of a point-mutated, non-cleavable subfragment of the *E. Coli* phage K1F endonuclease (endoNF) is known and a mechanism for the cleavage reaction has been proposed (Schulz et al., 2010). Cleavage occurs immediately to the N-terminal side of a conserved and essential serine residue (S911). The above results show that the site of cleavage of MrfA is approximately15 kDa away from the TM domain. It therefore approximately maps to the serine residue in MrfA (S767) equivalent to the endoNF cleavage site; as identified by the red arrow in Fig. 1B.

**Fig. 4.**
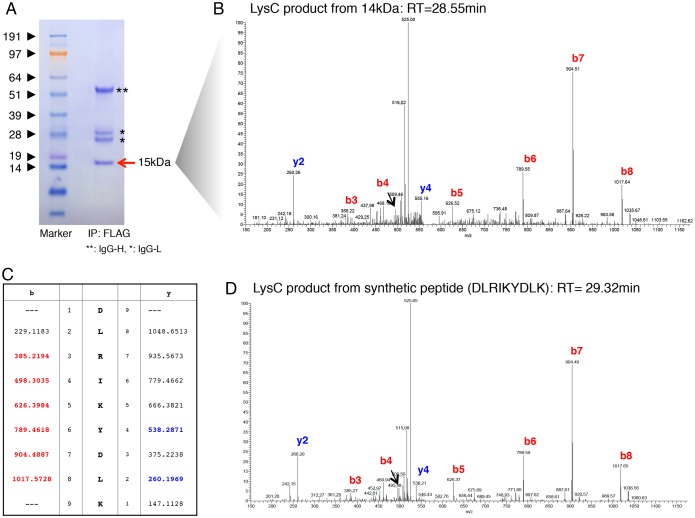
**Deduction of the cleavage site of MrfA.** (A) Purification of the C-terminal MrfA fragment. The C-terminal FLAG-tagged peptide of ∼14 kDa that derives from Myc-MrfAΔTM-FLAG was purified by immunoprecipitation with an anti-FLAG antibody and SDS-PAGE. The gel was stained with Coomassie Blue and the band excised. Two such preparations were made and both gave identical results in LC-MS/MS. (B) LC-MS/MS analysis of the C-terminal MrfA fragment. The 14 kDa gel slice was incubated with LysC and the solubilised peptides were separated by HPLC coupled to a mass spectrometer. There was no match to the species expected for complete LysC digestion of an MrfA cleavage product with S767 at its N-terminus (i.e. SDLRIK). There was, however, a product consistent with incomplete cleavage at K772, of an MrfA cleavage product with D767 at its N-terminus (i.e. DLRIKYDLK). (C) Depiction of the ion coverage in DLRIKYDLK This table shows the six b-series and the two y-series ions that closely match those predicted for an MS/MS analysis of peptide DLRIKYDLK. (D) LC-MS/MS analysis of the synthetic DLRIKYDLK peptide This is an analysis of a synthetic peptide with the predicted sequence identified in B (DLRIKYDLK). This showed a very similar retention time on HPLC to the immunopurified species and a very closely overlapping set of MS/MS peaks, including those that had not been assigned to the peptide sequence in B and C.

A more precise position for the cleavage site of MrfA was established by mass spectrometry, using as the start point cells expressing the TM deletion mutant: Myc-MrfAΔTM-FLAG. This was chosen because the 15 kDa, C-terminal fragment was present in much higher abundance than was observed with the 19 kDa parental form (Fig. 3B). The 15 kDa fragment was purified by immunoprecipitation and subjected to cleavage with LysC (Fig. 4A), which cleaves at the C-terminal side of lysine residues. The resultant peptides were subjected to liquid chromatography-tandem mass spectrometry (LC-MS/MS). There was no detectable peptide in the LC-MS/MS data, consistent with an N-terminus at S767 and cleavage downstream of the first lysine residue at the sequence SDLRIK. However, there was a peptide consistent with an N-terminus at D768 and incomplete cleavage by LysC at the sequence DLRIKYDLK (Fig. 4B,C). The presence of this peptide was confirmed by comparing the products derived from the immunopurified peptide with those from a synthetic peptide with the sequence DLRIKYDLK (Fig. 4D). This ostensibly maps the cleavage site to within one residue (i.e. S767) of the cleavage site of endoNF. However, because of the sequence similarity and because of the mutation analyses presented below, we believe that cleavage is at N-terminus of S767, but that there is an exopeptidase in the intact cell, within cell lysates or present during the immunoprecipitation, that digests S767. If there is indeed exopeptidase cleavage nearer to the N-terminus than S767, then we cannot be certain as to the position of the auto-proteolysis; we can only be certain that it lies somewhere upstream of D768. Aside from the sequence identity of the MRF domains, our evidence for cleavage at S767 derives from functional studies of mutated forms of MrfA.

### The MRF domain has functional similarity to bacteriophage autocatalytic cleavage domains

To test the functional significance of the sequence similarity, the predicted cleavage site in MrfA (S767) was mutated to alanine, to generate construct Myc-MrfA(S767A)-FLAG. The effect of the point mutation, when probing with the Myc antibody, was to generate a single species of ∼100 kDa; the approximate size expected for the unprocessed precursor (Fig. 3A). Probing with the FLAG antibody confirmed this by showing that the 19 kDa C-terminal fragment was absent and that there was again a major product of the approximate size expected for the unprocessed precursor (100 kDa). There were again additional bands of smaller size. These are presumably cleavage products, which retain the FLAG tag but lack the Myc tag and so are only detected with the FLAG antibody.

The S767A mutation was also inserted into the TM deletion form to generate Myc-MrfA(S767A)ΔTM-FLAG. This gave qualitatively similar results to the parental MrfA construct (Fig. 3B). As was noted above, the C-terminal FLAG-tagged product observed with the TM deletion form was much more abundant than with the parental form. Hence, its total absence using the S767A-ΔTM double mutant strengthens the case for the S767A mutation causing a complete block to cleavage.

We tested the analogy between the CIMCD and MrfA cleavage mechanisms further by mutating K772, the equivalent of K916 in endoNF CIMCD, to alanine. The resultant constructs Myc-MrfA(K772A)-FLAG and Myc-MrfA(K772A)ΔTM-FLAG showed an identical pattern of products to the corresponding S767A cleavage site mutants (Fig. 3A,B). Thus, both component parts of the serine-lysine dyad are essential for endo-proteolytic cleavage.

### Biochemical fractionation indicates that the two point mutant, uncleavable forms of MrfA co-purify with microsomes

**Fig. 5.**
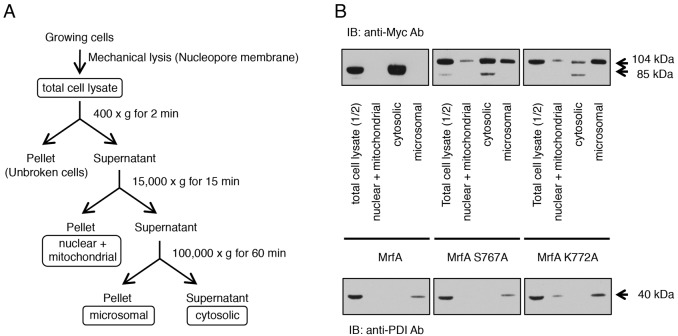
**Biochemical fractionation of MrfA and its point mutant forms.** Myc-MrfA-FLAG cells and mutant forms were subjected to lysis and biochemical fractionation as described in Materials and Methods. (A) The fractionation scheme. (B) Western blot analysis of the fractions. To compensate for expected losses during fractionation, only half of the total cell lysate was analysed. This, and the biochemical fractions, were analysed by western blotting. The resultant membrane was cut into a low molecular mass region that was probed for the classical ER marker PDI using a mixture of five monoclonal antibodies directed against different regions of the protein. The higher molecular mass region of the same filter was probed for Myc.

A differential centrifugation scheme was used to analyse the location of Myc-tagged MrfA in growing cells (Fig. 5A). The ER marker, protein disulphide isomerase (PDI), was analysed on the high-mobility part of the resultant western blot membrane to validate the fractionation. There was a sporadic low level of contamination of the nuclei and mitochondria fraction with PDI, but most of the PDI was, as expected, in the microsomal pellet (Fig. 5B). Myc-tagged MrfA, localised in the lower mobility part of the same membrane, is correctly cleaved during growth but the 85 kDa fragment accumulates exclusively in the cytosol; it presumably exits the microsomes but, because the analysis is conducted in growing cells, it fails to move to the nucleus. Analysis of the two point mutants supports this notion, because a significant proportion of the uncleaved precursor accumulated in the microsomal pellet (Fig. 5B). There was also a variable fraction of the mutant MrfAs in the cytosolic fraction so the overlap was not complete; perhaps because the tagged MrfA is overexpressed.

### Immunolocalisation of tagged MrfA and its mutant forms

**Fig. 6.**
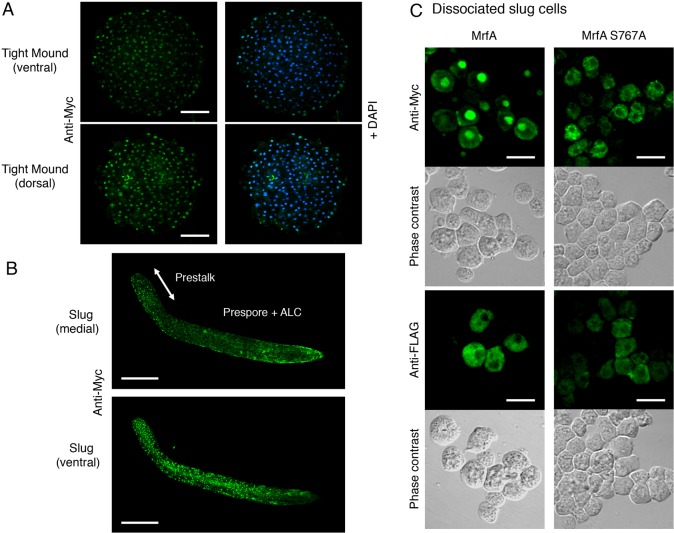
**Immunolocalisation of tagged MrfA in whole-mount and dissociated samples.** To localise MrfA, cells transformed with Myc-MrfA were analysed by whole-mount immunostaining and confocal microscopy at different developmental stages using the Myc antibody. (A) During growth, there is no nuclear accumulation of MrfA. During development, a gradually increasing proportion of cells display nuclear enrichment, such that by the tight-mound stage, all cells show nuclear staining. As the tip on the mound forms and extends, nuclear staining is lost from the emerging prespore region (data not shown). (B) In migrating slugs, the pattern of nuclear staining is partially dependent upon the duration of migration. In the medial region of newly migrating slugs, as identified by serial confocal sectioning, nuclear translocation occurs selectively in the prestalk region. In a ventral confocal section from the same slug, there is also nuclear enrichment in cells scattered throughout the slug. In older slugs, staining in the tip is often diminished but the scattered ventral staining remains. (C) Immunostaining of disaggregated slug cells expressing Myc-MrfA-FLAG or its S767A mutant derivative. *mrfA*^–^ cells, transformed with the indicated constructs, were developed to the slug stage and mechanically disaggregated. The cells were then fixed and stained with the 9E10 Myc antibody or the 1E6 FLAG antibody. The interpretation, based on correlating the western blot and Myc immunostaining results is as follows for MrfA and its S767A form (it is also true for the K772A form, data not shown). Myc-MrfA-FLAG (‘MrfA’) cells stained with Myc antibody show strong nuclear localisation in a subset of cells. Correlating this with the western blot results, indicates that the free 85 kDa product is efficiently translocated to the nucleus. With the point mutant, however, there is little or no nuclear accumulation of the 104 kDa, uncleaved form, presumably because it remains embedded in membrane. Since MrfA is predominantly present in its processed (85 kDa) form (Fig. 3A) in MrfA WT, FLAG staining is presumably directed to the residual (19 kDa) membrane-associated C-terminal fragment. There is a cytoplasmic meshwork of staining, with some degree of perinuclear localisation, but this was not analysed further. As expected from the total block to its proteolytic processing, the point mutant shows a similar FLAG staining pattern to that observed with the Myc antibody. Scale bars: 25 µm (A), 100 µm (B), 10 µm (C).

To localise MrfA more precisely, cells expressing the tagged, parental and mutant proteins under the control of an actin-15 promoter were used in immunostaining. In whole mounts at the tight mound stage of development all cells showed nuclear accumulation of Myc-MrfA (Fig. 6A). In whole mounts of newly migrating slugs, despite the fact that a semi-constitutive promoter is directing Myc-MrfA-FLAG transcription, only the prestalk cells and dispersed, ventrally located cells within the prespore zone showed nuclear Myc staining (Fig. 6B). Selective accumulation in the nuclei of prestalk cells accords well with MrfA being an activator of pstA-specific gene expression, whereas ventral expression of prestalk markers, in cells in the prespore zone is a common anatomical feature. Such cells form one class of ‘anterior-like cells’ (ALCs) (Sternfeld and David, 1982).

**Fig. 7.**
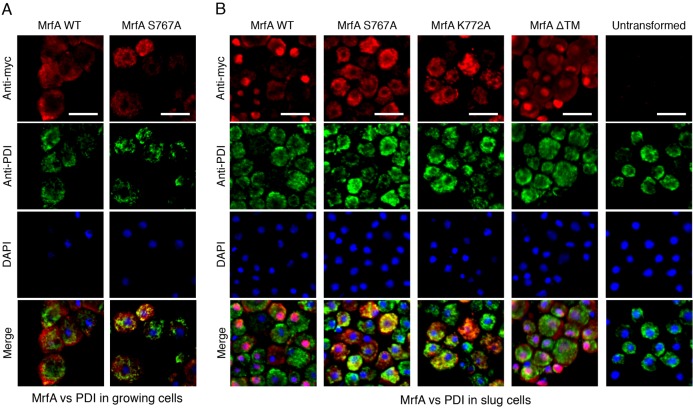
**Double immunostaining for Myc tagged MrfA and the ER marker PDI.** *mrfA*^–^ cells, transformed with the indicated constructs or left untransformed were analysed during growth (A) or developed to the slug stage (B), and partially mechanically disaggregated. After fixation, cells were stained with a mixture of a rabbit Myc antibody (red signal) and the cocktail of five monoclonal antibodies (green signal) directed against PDI. Nuclei were stained using DAPI (blue signal). In each field, a variable proportion of cells show typical ER staining (green) using the PDI antibody. The untransformed cells show no signal with the Myc antibody. In MrfA WT slug cells (cells transformed with Myc-MrfA-FLAG) there is strong nuclear staining with the Myc antibody in a subset of cells (red or sometimes purple signal, because of the overlap with DAPI staining). In cells transformed with the two point mutants and subjected to double staining for Myc and PDI, MrfA remains unprocessed and the product often colocalises with PDI (yellow or orange signal). In Myc-MrfAΔTM, deletion of the TM is permissive for processing and presumably liberates the 85 kDa fragment of MrfA from membranes. Consistent with this, Myc antibody, which detects the 85 kDda fragment (Fig. 3B), reveals nuclear staining. FLAG antibody, which detects the 15 kDda fragment (Fig. 3B), reveals patchy cytoplasmic staining (data not shown). Scale bars: 10 µm.

**Fig. 8.**
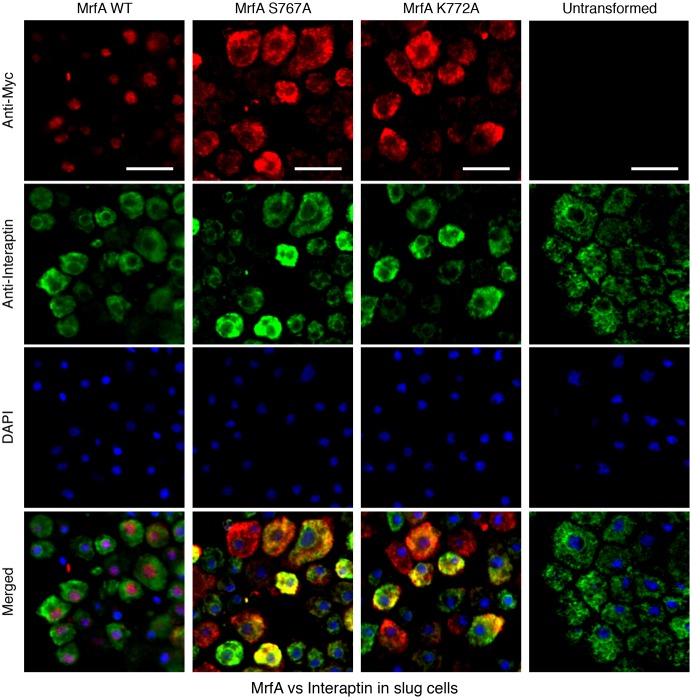
**Double immunostaining for Myc-tagged MrfA and interaptin.** *mrfA*^–^ cells, transformed with the indicated constructs or left untransformed, were developed to the slug stage and partially mechanically disaggregated. After fixation they were stained with a mixture of a rabbit Myc antibody (red signal) and a mouse monoclonal antibody (green signal) directed against interaptin. Nuclei were stained using DAPI (blue signal). In each field, a variable proportion of cells show typical ER staining (green) using the interaptin antibody. The untransformed cells show no signal with the Myc antibody. In MrfA WT cells (cells transformed with Myc-MrfA-FLAG) there is strong nuclear staining with the Myc antibody in a subset of cells (red or sometimes purple signal, because of the overlap with DAPI staining). In cells transformed with the two point mutants and subjected to double staining for Myc and interaptin, MrfA remains unprocessed and the product often colocalises with interaptin (yellow or orange signal). Scale bars: 10 µm.

When slug cells transformed with Myc-MrfA-FLAG were dissociated and stained with Myc antibody (green fluorescence) only a proportion of cells, most often those within clumps, showed nuclear staining (Fig. 6C). The likely reason for heterogeneity between microscopical fields, is the selective nuclear accumulation of MrfA in specific subtypes of prestalk cell (Fig. 6B). The point mutations in the serine-lysine dyad that completely block proteolytic processing had an equally profound effect on nuclear localisation; with the two point mutants there was little or no Myc staining in the nucleus. Instead staining occurred in a subset of cells (Fig. 6C; Figs 7,8; Table 1). In these cells, there were strongly staining punctae in the cytoplasm and a perinuclear ring could often be discerned. By contrast, analysis of the TM deletion mutant Myc-MrfAΔTM-FLAG using the Myc antibody yielded a pattern very similar to that of the parental MrfA form, with a fraction of slug cells displaying intranuclear enrichment (Fig. 7B, Table 1). This is also as expected from the western blot analysis, which shows that possession of a TM domain is not essential for correct processing to yield the 85 kDa fragment (Fig. 3B). There is, however, developmental regulation of the nuclear accumulation of the N-terminus proximal 85 kDa fragment of MrfA; because, despite the fact that the cleavage occurred with high efficiency in growing cells (Fig. 3A), there was little or no nuclear accumulation (Fig. 7A,B; Table 1).

**Table 1.**
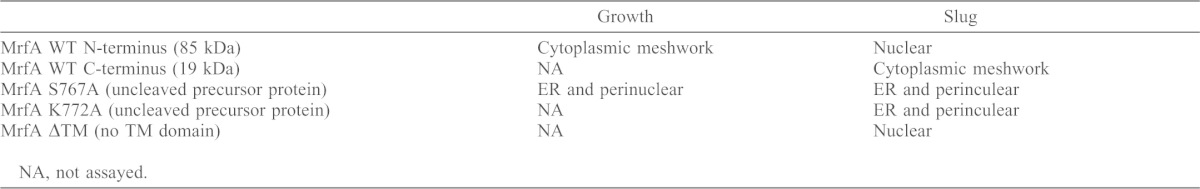
Intracellular localisation of MrfA and its mutant forms in growing and developing cells

NA, not assayed.

### The uncleavable mutant forms of MrfA reside in the ER

The above results suggest that the two point-mutated proteins accumulate in the ER. This was confirmed by double immunostaining of vegetatively growing cells or disaggregated slug cells, again doubly expressing Myc- and FLAG-tagged MrfA under the control of the actin-15 promoter. Localisation of the Myc fusion protein was analysed using a rabbit anti-Myc polyclonal antibody (red fluorescence). The ER was localised by detecting PDI and also interaptin, a developmentally regulated ER marker that is a member of the α-actinin family (Rivero et al., 1998).

The images for PDI at both the vegetative and the slug stages, revealed quantitatively heterogeneous staining between cells (Fig. 7A,B). However, the distribution was similar, with granular staining adjacent to the nuclear membrane. With the N-terminally Myc-tagged MrfA fragment, there was also quantitative heterogeneity of Myc staining at both stages. This was as expected, because the fusion protein is expressed under the control of the developmentally regulated actin-15 promoter and there will be variation in its copy number. Despite this variation in intensity of staining, the merged images revealed a fraction of growing and slug-stage cells where PDI colocalised with the S767A and K772A mutant forms of MrfA (Fig. 7A,B; Table 1). The major difference between growth- and slug-stage cells was that the Myc-tagged 85 kDa MrfA protein fragment remained in the cytoplasm of growing cells (Fig. 3A; Fig. 7A; Table 1) but accumulated in the nuclei of slug cells (Fig. 7A; Table 1). We also analysed slug cells expressing the doubly tagged MrfA mutant that lacks the TM domain (Myc-MrfAΔTM-FLAG). As expected from the fact that it is processed correctly to yield an 85 kDa fragment (data not shown), the Myc-tagged fragment accumulated in slug nuclei (Fig. 7B).

In an attempt to increase the degree of overlap at the slug stage, we also analysed interaptin, an ER marker that we suspected might better correspond in its expression pattern with those cells in which MrfA accumulates in the ER. The merged images did indeed reveal a higher proportion of double staining cells, with co-staining often localised to the perinuclear region (Fig. 8; Table 1). Again overlap was not complete because, although interaptin and MrfA are both prestalk specific, they have only partially overlapping subtype specificities; in whole mounts of slugs, interaptin predominantly localises to a subset of the ALCs that are scattered to the rear of the prespore region (Rivero et al., 1998). MrfA was expressed in anterior prestalk cells and ventrally located ALCs (Fig. 6B). Presumably, the latter are the cells in which PDI colocalises with the S767 and K772 mutant forms of MrfA.

We conclude that MrfA is synthesised as a membrane-tethered protein that is initially localised in the ER. Analysis of dissociated slug cells expressing the parental construct using FLAG antibody supports this interpretation because the 19 kDa, C-terminally FLAG-tagged fragment of MrfA, detected by western blotting, was localised in a very similar manner to the two point-mutant forms of MrfA (Fig. 6C; Table 1) in cytoplasmic patches that were often perinuclear.

### Biological function requires cleavage at S767, but membrane insertion is not obligatory

**Fig. 9.**
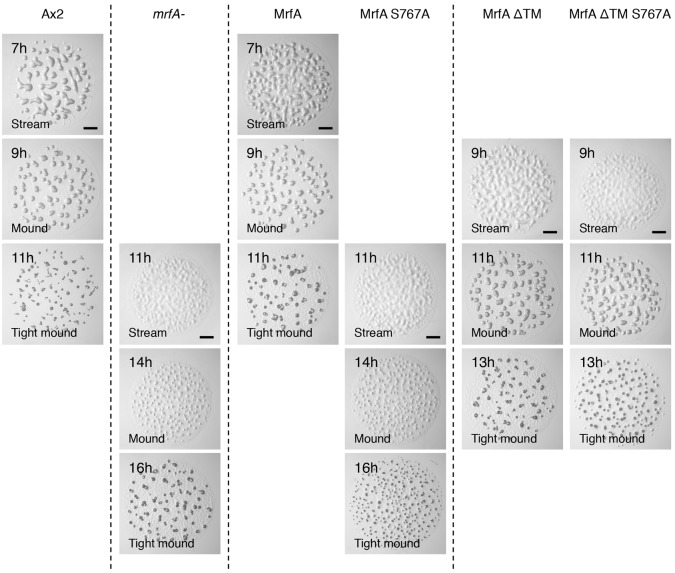
**Complementation of the *mrfA*^–^ phenotype with parental and mutant MrfA constructs.** The *mrfA*^–^ strain was left untransformed or transformed with the indicated constructs. Development was analysed on water agar plates, in parallel with the Ax2 parental strain. Scale bars: 100 µm.

The biological requirement for the various processing steps was assessed using the mutant forms of MrfA to complement the *mrfA* null strain. The *mrfA*^–^ phenotype had an approximate 4 hour delay in development (Fig. 9). Transformation of the *mrfA*^–^ strain by parental MrfA restored normal timing of development, whereas the two point-mutant forms (data not shown for MrfAK772A) failed to do so. Thus, cleavage of the membrane inserted 105 kDa form is necessary for normal biological function. This requirement could, however, be avoided if membrane insertion was blocked by deletion of the TM domain, because transformation with MrfAΔTM and its two point mutant derivatives all complemented the *mrfA*^–^ strain correctly.

## Discussion

### MrfA contains a predicted TM domain that is not necessary for cleavage

**Fig. 10.**
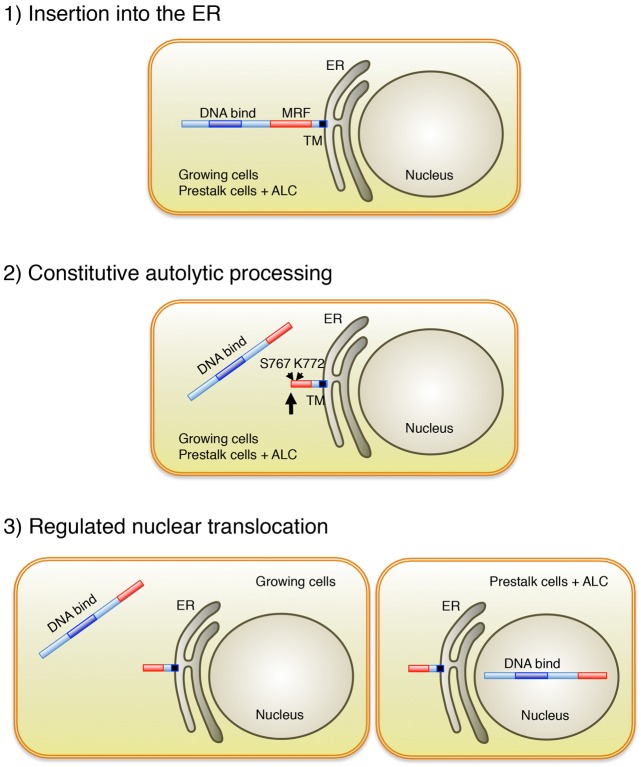
**Schematic illustrating the proposed processing pathways for MrfA.** MrfA is inserted into the ER by its C-terminus-proximal TM domain. It is then constitutively self-cleaved at Ser767. In growing cells, the liberated fragment remains cytosolic. In some prestalk cells and ALC the liberated fragment is activated and accumulates in the nucleus.

We show that the MRF protein family contain, as a sequence feature additional to the conserved DNA binding and MRF domains, a predicted TM domain. Apart from lacking the long simple repeat sequence tracts that are found in many *Dictyostelium* proteins, the animal MRFs principally differ from MrfA in possessing a much longer region C-terminal to the predicted TM domain. The function of this additional sequence is unclear. In the case of MrfA, we have demonstrated by double tagging, that an N-terminal fragment of ∼85 kDa and a C-terminal fragment of ∼19 kDa accumulate in growing and developing cells (Fig. 10). In developing (but not in growing) cells, the 85 kDa fragment accumulated in the nucleus, whereas the 19 kDa fragment was retained within the cytoplasm. In a C-terminal deletion mutant, lacking the TM domain, cleavage of the 104 kDa precursor and nuclear accumulation of the 85 kDa fragment both occurred efficiently. This is in complete contrast to the archetypal membrane-tethered transcription factors, which are released by cleavage within the plane of the ER membrane.

### MrfA contains a serine-lysine dyad that directs its cleavage

Whereas RIP occurs by cleavage catalysed by extrinsic proteases, cleavage of MrfA is an autocatalytic process. The evidence for this is in part bioinformatic but is, nonetheless, highly compelling. The MRF domain has sequence similarity to the CICMD of bacteriophage structural proteins. The CIMCD is an autocatalytic protease cleavage site. It is cleaved to the immediate N-terminal side of a serine residue: S911 in endoNF. When aligned with MrfA, the equivalent serine residue is S767, and this residue is essential for cleavage and for accumulation in the nucleus. The other essential component in the endoNF CIMCD cleavage reaction is the nearby lysine residue K916. It acts as a general base in a serine/lysine dyad mechanism, whereby the lysine removes a proton from the hydroxyl group of S911, allowing for its nucleophilic attack on the adjacent, scissile peptide bond (Schulz et al., 2010). Mutation of the equivalent lysine in MrfA also totally inhibits processing and nuclear accumulation. Interestingly, the residue at the N-terminal side of the cleavage position, the equivalent of T910 in the endoNF CIMCD, is not conserved with the MRFs (Fig. 1B). However, in the endoNF CIMCD, a hydrogen bond between T910 and N912 is proposed to cause a kink in the chain, generating a tension at the N-terminus of the CIMCD and rendering the bond scissile (Schulz et al., 2010). In the MRFs, the residue at the N-terminal side of the cleavage position is a conserved proline, which presumably subsumes the same chain-distorting function. Aside from this difference, the cleavage mechanism to be similar in bacteriophage CIMCDs and eukaryote MRFs. We therefore speculate that either: (1) it arose before the divergence of prokaryotes and eukaryotes, or (2) there was horizontal gene transfer from an ancestral prokaryote into a common ancestor of amoebozoans and metazoans.

### Nuclear accumulation of MrfA requires cleavage from the ER

In marked contrast to proteins subject to RIP, MrfA is not sequestered in the ER; rather it presumably suffers a rapid, autocatalytic cleavage. This would explain why we were able to detect only ER-associated MrfA when the serine-lysine dyad cleavage mechanism was mutationally inactivated. Why, if it is not sequestered there as part of a regulatory mechanism, does MrfA make this transient detour to the ER? Perhaps we can obtain an insight from the CIMCDs. They undergo auto-proteolysis that is triggered by the trimerisation of the tail-spike precursor molecules. Possibly, therefore, association of MRFs with the ER promotes their multimerisation and it is the act of multimerisation that triggers auto-proteolysis. In this respect, it is interesting to note that the sequence similarity between the MRF domain and the CIMCD extends well outside the core of residues directly involved in autocatalysis, supporting the notion of a common function, such as the mediation of a homotypic interaction.

### Distinct signalling pathways for different prestalk cell subtypes

At the slug stage, MrfA selectively accumulates in the nuclei of prestalk cells. This fits the known role of MrfA as an activator of pstA cell differentiation and leaves open the possibility of a function in pstO cells. Interestingly, at the preceding tight-mound stage, all cells show nuclear accumulation of Myc-MrfA. By contrast, despite the fact that proteolytic processing occurs efficiently in growing cells, there is no detectable nuclear accumulation in growing cells. In conjunction, these results again suggest that proteolytic processing is constitutive and is effectively unlinked from a developmentally regulated mechanism that controls nuclear accumulation. This latter mechanism, which becomes operative between the tight-mound and the slug stages, prevents processed Myc-MrfA-FLAG from accumulating in the nuclei of prespore cells and also of the prestalk cell subtypes other than pstA cells and a subset of the ventrally located ALCs.

Extracellular signalling in the prestalk cell types, other than the pstA cells, is partially understood. Cap-site distal elements direct a relatively low level of *ecmA* gene expression in the posterior half of the prestalk region – in the pstO cells (Early et al., 1993). There is also a population – the pstB cells – that cluster on the ventral surface of the prespore region of the slug near the prestalk boundary. They express *ecmA* at a relatively low level but can be recognised by their relatively high level expression of the closely related *ecmB* gene and by their selective staining with vital dyes (Dormann et al., 1996; Jermyn et al., 1996). Correct pstO-specific gene expression, and the formation of pstB cells, are both induced by DIF-1, a chlorinated hexaphenone secreted by the prespore cells (Thompson and Kay, 2000). However, the extracellular signal inducing pstA differentiation is probably not DIF-1 because mutants defective in DIF-1 synthesis and signalling still express *ecmA* in the pstA region (Thompson et al., 2004). In addition, we find that: (1) the pstA-specific *lacZ* reporter is not induced by DIF-1 in a monolayer assay; and (2) addition of DIF-1 to cells in early development (at 5 hours of starvation) does not increase the proportion of cells with Myc-tagged MrfA in the nucleus (unpublished data). These are negative results but, in conjunction with the genetic analyses, they focus the search for the extracellular signal that induces nuclear translocation of MrfA elsewhere: perhaps on a polyketide other than DIF-1 (Serafimidis and Kay, 2005).

## Materials and Methods

### Cell growth and development

*D. discoideum* Ax2 (Gerisch isolate) was used as the parental strain. Cells were grown in HL5 medium (Watts and Ashworth, 1970) and developed on water or KK2 (20 mM K_2_HPO_4_/KH_2_PO_4_ pH 6.2) agar or on membrane filters, at a density of 1.0×10^6^ cells/cm^2^. Migrating slugs were generated by developing cells on water agar under dim unidirectional light.

### Subcellular fractionation

Exponentially growing cells (WT, S767A and K772A) were harvested and washed twice in KK2 buffer. Cells were resuspended at a density of 1×10^8^ cells/ml in filter lysis buffer [10 mM Tris-HCl, pH 8.0, 250 mM sucrose, Complete Protease inhibitor cocktail (Roche)]. Cells were mechanically lysed by passing through a nucleopore membrane (pore size 3 μm, Millipore) three times. After removing unbroken cells by centrifugation at 400 ***g*** for 4 minutes at 4°C, the cell lysate was centrifuged at 15,000 ***g*** for 15 minutes at 4°C to collect the nuclei and mitochondria fraction (also containing plasma membranes). The clarified cell lysate was subjected to ultracentrifugation (100,000 ***g*** for 60 minutes at 4°C) to separate the soluble fraction and the microsomal fraction.

### Generation of doubly tagged forms of MrfA

To generate a Myc- and FLAG-tagged form of MrfA, the entire ORF was amplified using *mrfA* cDNA. The primers incorporated a *Cla*I site and a Myc epitope in the 5′ primer and a *Xho*I site and a FLAG epitope in the 3′ primer: MycClaI, atcgatGAACAAAAATTAATTTCAGAAGAAGATTTAAATAAAATGGATGGGTATAACCAACAGCAACAG; FLAGXhoI, ctcgagTTATTTATCATCATCATCTTTATAATCATTATCATTATTATCAAAATTATGAC.

After digestion with *Cla*I and *Xho*I, the fragment was cloned into *Cla*I- and *Xho*I-cut pA15GFPS65T (Heim et al., 1995) to generate Myc-MrfA-FLAG. An equivalent procedure was used to generate a Myc- and FLAG-tagged form of MrfAΔTM, except that the 3′ primer was designed to generate a termination codon at a point 38 codons upstream of the natural C-terminus. This generated Myc-MrfAΔTM-FLAG. To generate Myc- and FLAG-tagged forms of mutants S767A and K772A, point mutations were introduced into Myc-Mrf-FLAG or Myc-MrfAΔTM-FLAG, using the QuikChange mutagenesis kit (Stratagene): mrfA S767A antisense, CTTAAATCagcTGGATGATAAAC; mrfA S767A sense, GTTTATCATCCAgctGATTTAAG; mrfA K772A antisense, CGATTTCAAATCATAtgcTATTCTTAAATC; mrfA K772A sense, GATTTAAGAATAgcaTATGATTTGAAATCG (lowercase letters represent point-mutated residues).

### Western blot analysis

Proteins were separated on 4–12% SDS gels, blotted onto nitrocellulose membranes and probed with either an anti-Myc monoclonal antibody (9E10) or an anti-FLAG monoclonal antibody (1E6, Wako). Horseradish-peroxidase-coupled goat anti-mouse IgG secondary antibodies were used, with a final ECL detection.

### Immunochemistry

Transformant cells were fixed in cold methanol and either stained with anti-Myc monoclonal antibody, 9E10 or, for double staining, rabbit anti-Myc polyclonal antibody. Interaptin was detected in double staining with Myc using mAb 260-60-10 (Rivero et al., 1998; a gift from Angelika Noegel, Institute of Biochemistry, University of Cologne), followed by Alexa-Fluor-488-conjugated goat anti-mouse IgG and Alexa-Fluor-594-conjugated goat anti-rabbit IgG (Invitrogen). FLAG staining was performed using monoclonal antibody 1E6 followed by Alexa-Fluor-488-conjugated goat anti-mouse IgG. Nuclei were visualised in the double immunostaining experiments using 4′,6-diamidino-2-phenylindole (DAPI; Sigma). PDI was detected with an antibody mix supplied by Thierry Soldatti (Dept of Biochemistry, University of Geneva) and used as described previously (Monnat et al., 1997).

### Immunoprecipitation and mass spectrometry

5.0×10^8^ vegetative cells expressing Myc-MrfAΔTM-FLAG were lysed in 1 ml of mNP40 lysis buffer [50 mM Tris-HCl, pH 8.0, 150 mM NaCl, 1% (v/v) Nonidet P-40, 50 mM NaF, 2 mM EDTA, pH 8.0, 2 mM Na-pyrophosphate and complete EDTA-free protease inhibitor mixture (Roche Diagnostics] for 10 minutes on ice. After pre-clearing by centrifugation, the supernatant was incubated with anti-FLAG antibody for 30 minutes at 4°C with gentle agitation, followed by another 2 hours of incubation with Dynabeads Protein-G (Life Technologies). Beads were washed four times in mNP40 buffer, then bound proteins were eluted by boiling in SDS gel sample buffer and concentrated by acetone precipitation. For mass spectrometry, the re-solubilised sample was loaded in its entirety on a 12% Bis-Tris NuPAGE gel with MOPS buffer (Invitrogen) and stained with Colloidal Blue (Invitrogen). The 15 kDa band was excised and subjected to in-gel digestion with LysC and LC-MS/MS analysis. Samples were reductively alkylated with DTT and IAA then digested with LysC overnight and the peptides extracted. Aliquots were run on an LTQ Orbitrap Velos Pro (Thermo Scientific) system coupled to an RSLC nano HPLC system (Thermo Scientific/Dionex) under the following conditions. The peptides were loaded onto a Thermo/Dionex trap column (Acclaim PepMap 100, 100 µm × 2 cm nanoViper, C18, 5 µm) and eluted using a linear gradient over 65 minutes of 2–40% buffer B (80% acetonitrile, 0.08% formic acid; Buffer A, 0.1% formic acid) onto a Thermo/Dionex nano column (Acclaim PepMap RSLC, 75 µm × 15 cm nanoViper, C18, 2 µm). The eluent from the nano column was introduced into the mass spectrometer through a New Objectives PicoTip emitter (SilicaTip, FS 360-20-10-N-20-C12). The LTQ Orbitrap Velos Pro used a TOP15 method [1× FT-MS at 60,000 resolution with lock mass (445.120024) followed by 15× IT-MSMS scans for peptide fragmentation]. Analysis of the data was performed using Proteome Discoverer (Ver. 1.3) along with the Mascot Search Engine (Ver. 2.3.2).
